# Advancing fracture risk prediction: established assessment tools and emerging HR-pQCT-based approaches

**DOI:** 10.3389/fendo.2026.1916830

**Published:** 2026-10-05

**Authors:** Raveen K. Sidhu, Meghan G. Donaldson, Steven K. Boyd

**Affiliations:** 1 McCaig Institute for Bone and Joint Health, Cumming School of Medicine, University of Calgary, Calgary, AB, Canada; 2 Department of Biomedical Engineering, Schulich School of Engineering, University of Calgary, Calgary, AB, Canada; 3 Department of Radiology, Cumming School of Medicine, University of Calgary, Calgary, AB, Canada

**Keywords:** bone, bone microarchitecture, computed tomography, fracture prediction, fracture risk assessment, HR-pQCT, osteoporosis, falls

## Abstract

Osteoporotic fractures, or fragility fractures, pose a substantial global healthcare burden. Individuals who experience them report significant declines in their quality of life. Identifying individuals at risk of fragility fractures is key to reducing the future fracture burden. However, due to demographic shifts, the proportion of older adults is growing globally. As fracture risk rises with age, an increase in the incidence of osteoporotic fractures is also anticipated. However, existing screening initiatives and fracture risk assessment tools remain challenged in identifying individuals at risk of experiencing osteoporotic fractures. To adapt to the predicted incidence of fragility fractures, knowledge of established fracture risk tools and emerging approaches is needed to inform the development of future fracture prediction tools. Correspondingly, this narrative review presents an overview of established fracture risk assessment tools, including FRAX, FRAXplus, CAROC, Garvan, and QFracture, which use clinical risk factors and frequently, bone mineral density estimates, to produce fracture risk prediction scores. We review methods, advantages, limitations, and performance of the established tools in detail. Later, we highlight emerging fracture risk assessment tools that integrate direct assessment of bone using high-resolution peripheral quantitative computed tomography (HR-pQCT). The advantages and limitations of the emerging HR-pQCT-based fracture risk assessment models are also reviewed in detail. Finally, future directions for fracture risk assessment tool development are discussed.

## 1 Introduction

Osteoporosis is an age-related degenerative bone disease where declines in bone microarchitecture and mass contribute to an increased risk of fracture (1, 2). Clinically, osteoporosis is defined as a bone mineral density (BMD) of 2.5 or more standard deviations below peak bone mass (i.e., T-score ≤ -2.5) (3). Osteoporosis may also be diagnosed in individuals aged 50 or greater if they have experienced a low-trauma hip, vertebral, humerus, or pelvic fracture after the age of 40, or if an individual receives an absolute fracture risk score exceeding 20% over the next 10 years as assessed with tools like FRAX (discussed later) (3). The deterioration in bone tissue arises due to an uncoupling between bone building (mediated by osteoblasts) and bone resorption processes (mediated by osteoclasts) (1, 2). In young, healthy adults, bone resorption and formation are nominally balanced. During aging, bone deposition mechanisms are impaired, resulting in a net decrease in bone material and microarchitecture (2). This produces an overall degeneration of bone and increased susceptibility to fracture (2, 4). Adults over the age of 65 demonstrate a substantially increased fracture risk compared to younger adults (5), with many of these fractures associated with osteoporosis.

A major consideration when managing patients with osteoporosis is minimizing the risk of fracture (6). Attention to minimizing fracture risk is important because individuals who experience an osteoporotic fracture are likely to suffer a significant reduction in quality of life (7–9), mobility (10–13), independence (10–13), mental wellbeing (14, 15), financial hardship (16), and are at increased risk of mortality following fracture (17, 18). Given the substantial burden of osteoporotic and subsequent fractures, there is motivation to identify at-risk individuals earlier and intervene before fractures occur. In addition to the negative outcomes presented by osteoporosis at the patient level, the societal burden of osteoporotic fractures is immense. Excluding costs associated with disability and productivity loss, the direct cost of treating osteoporotic fractures in the European Union alone was estimated at €37 billion in 2010 and was projected to rise by 25% by 2025 (19). Annual costs are predicted to increase due to changing population demographics as the number of aging adults increases, resulting in a higher portion of the population afflicted with osteoporosis (19, 20). As such, the identification of individuals at the greatest risk of fracturing is important (21).

Despite the growing societal burden of fragility fractures, existing fracture risk assessment tools have limited sensitivity and specificity (22). Many individuals classified as having either osteopenic (i.e., low bone mass) or normal bone health as identified by current clinical tools still go on to experience fragility fractures (23–25), highlighting the issue of false negatives. Advances in imaging resolution now enable direct assessment of bone quality and microarchitecture, however most fracture risk assessment tools continue to incorporate clinical risk factors (CRFs) as indirect proxies of bone health. Leveraging bone imaging advancements may improve fracture risk prediction by providing bone microarchitectural information (26). Improving fracture risk assessment tools may enable earlier intervention through appropriate pharmacologic treatment and lifestyle modifications, which may subsequently reduce the occurrence of fragility fractures.

This narrative review begins with an overview of existing fracture risk assessment tools which rely on indirect assessment of bone using areal BMD (aBMD) and CRFs, followed by a discussion on the strengths and limitations of these tools. Next, we review emerging fracture risk assessment tools, particularly focusing on those that integrate a direct assessment of bone quality using high-resolution peripheral quantitative computed tomography (HR-pQCT). We present strengths and limitations of these HR-pQCT-based fracture risk assessment tools. Finally, we discuss future directions toward the improvement of fracture risk assessment.

### 1.1 Established tools in fracture risk prediction

A widely used approach in fracture risk assessment involves bone mineral density (BMD) based on areal measurements (usually denoted as aBMD), obtained using dual energy X-ray absorptiometry (DXA) and the use of a normative reference database. While low BMD is an established risk factor for both hip fracture and non-hip fracture (24, 27), the majority of fractures occur in individuals with BMD T-score > -2.5, limiting its ability to identify individuals at risk of fracture (28). The suboptimal performance of aBMD measures to identify individuals with increased fracture risk has fostered the development of risk prediction tools that integrate CRFs to enhance risk stratification (**Table 1**) (28). Common fracture risk assessment tools have demonstrated predictive performance comparable to or exceeding that of BMD alone by integrating CRFs with aBMD within logistic and Cox proportional hazards models. We discuss the strengths, performance, and limitations of the most prevalent fracture risk identification tools: DXA, FRAX and FRAXplus, CAROC, Garvan, and QFracture.

**Table 1:** Fracture risk prediction tools: clinical risk factors and reported outcomes.

Access	FRAX	FRAXplus	CAROC	Garvan	QFracture-2012, QFracture-2016^*^	μFRAC^a^
Cost	No cost	0–4,000 €/year (usage-dependent)	No cost	No cost	No cost	No cost
Website	fraxplus.org/calculation-tool	fraxplus.org/frax-plus	osteoporosis.ca/wp-content/uploads/CAROC.pdf	fractureriskcalculator.com.au/calculator	qfracture.org	normative.ca

Bone-specific factors	FRAX	FRAXplus	CAROC	Garvan	QFracture-2012, QFracture-2016^*^	μFRAC^a^
aBMD, Femoral neck	X (optional; aBMD in g/cm^2^ or T-score)	X (optional; aBMD in g/cm^2^ or T-score)	X (T-score)	X (optional; aBMD in g/cm^2^ or T-score)		X (optional^a^; aBMD in g/cm^2^)
Fracture history; general	X	X (recency, site, and number of previous fractures, input as 0, 1, 2, 3, 4 or more)			X (specific to wrist, spine, hip, or shoulder fractures)	X (adulthood fracture, optional)
Fracture history; parental history of hip fracture	X	X			X (grouped with parental history of osteoporosis)	
Fracture history; osteoporotic			X (after age 40)	X (no. after age 50, input as 0, 1, 2, 3 or more)		
Microarchitecture		X (optional, estimated through TBS)				X (HR-pQCT density and microarchitecture parameters^b^)
aBMD, Lumbar spine		X (optional; adjustment for differences between lumbar-spine and femoral-neck T-scores)				
Hip axis length (in millimeters, mm)		X (optional)				
Microfinite element analysis; failure load (N)						X (optional)

Fall-specific factors	FRAX	FRAXplus	CAROC	Garvan	QFracture-2012, QFracture-2016^*^	μFRAC^a^
Number of falls in the past 12 months; risk gradient		X (no. input as 0, 1, 2, or 3+)		X (no. input as 0, 1, 2, or 3+)		
Fall history; binary yes/no					X	

Clinical risk factors	FRAX	FRAXplus	CAROC	Garvan	QFracture-2012, QFracture-2016^*^	μFRAC^a^
Age (years)	X	X	X	X	X	X
Sex	X	X	X	X	X	X (optional)
Weight (kg)	X (used to calculate BMI)	X (used to calculate BMI)		X (if aBMD is not provided)	X (optional; used to calculate BMI)	X (optional)
Height (cm)	X (used to calculate BMI)	X (used to calculate BMI)			X (optional; used to calculate BMI)	X (optional)
Glucocorticoids	X (current oral use or previous use >3 months at ≥5 mg/day prednisolone equivalent)	X (FRAX definition, with dose-based adjustment)	X (≥7.5 mg/day prednisone equivalent for ≥3 months in past year)		X	
Ethnicity	X (country/model-dependent)	X (country/model-dependent)			X (optional)	
Alcohol intake	X (3 units or more/day)	X (3 units or more/day)			X (categories of exposure)	
BMI (kg/m^2^)	X	X			X (optional)	
Chronic liver disease	X (grouped under secondary osteoporosis)	X (grouped under secondary osteoporosis)			X	
Chronic renal disease	X (chronic renal failure (dialysis independent), grouped under secondary osteoporosis)	X (chronic renal failure (dialysis independent), grouped under secondary osteoporosis)			X (Yes, if stage 4 or 5)	
Diabetes	X (type 1 diabetes, grouped under secondary osteoporosis)	X (years since type 2 diabetes diagnosis; type 1 diabetes is grouped under secondary osteoporosis)			X (input as none, type 1, type 2)	
Endocrine conditions	X (untreated long-standing hyperthyroidism, hypogonadism or premature menopause (<45 years), grouped under secondary osteoporosis)	X (additional adjustment for primary hyperparathyroidism; untreated long-standing hyperthyroidism, hypogonadism or premature menopause (<45 years) grouped under secondary osteoporosis)			X (grouped with thyrotoxicosis, hyperparathyroidism, Cushing’s syndrome)	
Malabsorption conditions	X (chronic malnutrition or malabsorption, grouped under secondary osteoporosis)	X (chronic malnutrition or malabsorption, grouped under secondary osteoporosis)			X (Grouped with Crohn’s disease, ulcerative colitis, coeliac disease, steatorrhea or blind loop syndrome)	
Rheumatoid arthritis	X	X			X (grouped with systemic lupus erythematosus (SLE))	
Secondary osteoporosis	X (Yes/No if any of these conditions are present: type 1 diabetes, osteogenesis imperfecta in adults, untreated long-standing hyperthyroidism, hypogonadism or premature menopause (<45 years), chronic malnutrition or malabsorption, chronic renal failure (dialysis independent), or chronic liver disease)	X (Yes/No if any of these conditions are present: type 1 diabetes, osteogenesis imperfecta in adults, untreated long-standing hyperthyroidism, hypogonadism or premature menopause (<45 years), chronic malnutrition or malabsorption, chronic renal failure (dialysis independent), or chronic liver disease)			X (covered by separately listed conditions; no standalone input)	
Smoking status	X	X			X (categories of exposure)	
Antidepressants					X	
Asthma or chronic obstructive pulmonary disease (COPD)					X	
Cancer					X	
Cardiovascular Disease					X (heart attack, transient ischemic attack, angina, or stroke)	
Dementia					X	
Epilepsy					X (Yes, if epilepsy or taking anticonvulsants)	
Hormone replacement therapy (HRT)					X (oestrogen-only HRT)	
Nursing/care home residence					X	
Parkinson’s disease					X	

Model output	FRAX	FRAXplus	CAROC	Garvan	QFracture-2012, QFracture-2016^*^	μFRAC^a^
10-year risk of Major Osteoporotic Fracture, (MOF)^c^	X	X	X (risk categories; outputs 10-year risk as low, moderate, or high)		X (available at 1–10-year intervals in the most recent QFracture model)	X
5-year risk of MOF^c^					X (available at 1–10-year intervals in the most recent QFracture model)	X
10-year risk of hip fracture	X	X		X	X (available at 1–10-year intervals)	
5-year risk of hip fracture				X	X (available at 1–10-year intervals)	
10-year risk of Garvan Any Osteoporotic Fracture, (AOF)^d^				X		
5-year risk of Garvan AOF^d^				X		
10-year risk of μFRAC AOF^e^						X
5-year risk of μFRAC AOF^e^						X

Clinical risk factors (CRFs) are binary yes/no inputs unless otherwise indicated, with categorical exceptions being sex and ethnicity. Within each section, factors are ordered from most to least frequently included across the risk prediction tools. Bone-related and fall-related risk factors are grouped separately.

^*^QFracture-2012 and QFracture-2016 share the same CRF and model output categories, allowing them to be grouped within a single column. However, the statistical weights of CRFs differ between the two models.

^a^Depending on input availability, one of three μFRAC model variants can be used. The complete μFRAC model uses inputs of age, standard HR-pQCT parameters, sex, height, weight, adulthood fracture history, FEA failure load, and FN.aBMD. μFRAC′ requires age, standard HR-pQCT parameters, and FEA failure load. μFRAC″ requires only age and standard HR-pQCT parameters. For readability, these tool variations have been grouped into one column.

^b^Standard HR-pQCT parameters include measurements at the distal radius and tibia of total, cortical, and trabecular volumetric BMD (mg HA/cm³); total, cortical, and trabecular cross-sectional areas (mm^2^); derived cortical thickness (mm); trabecular number (mm^-^¹); and trabecular inhomogeneity (mm).

^c^MOF includes the hip, spine, wrist, or shoulder.

^d^Garvan Any Osteoporotic Fracture (AOF*)* is defined as fractures of the hip, wrist, metacarpals, humerus, scapula, clavicle, proximal tibia, pelvis, sternum, distal femur, and patella, and symptomatic vertebral fractures.

^e^μFRAC Any Osteoporotic Fracture (AOF) is defined as the first incident non-traumatic fracture of the shoulder/collar, upper arm, elbow, upper forearm, wrist/lower forearm, hand, rib, spine, coccyx, pelvis, hip, lower thigh, knee, shin, ankle, foot, and heel. μFRAC AOF explicitly excludes high-trauma fractures, pathologic fractures, and radiographic vertebral fractures that are not clinically detected.

The tools selected for review were informed by major national and international osteoporosis clinical guidelines including: the World Health Organization (WHO) and the International Osteoporosis Foundation (IOF) for DXA (29, 30); Osteoporosis Canada for CAROC (3); Osteoporosis New Zealand and Healthy Bones Australia for the Garvan calculator (31–33); and the UK National Institute for Health and Care Excellence (NICE) for QFracture (30). Notably, FRAX is recommended by more than 120 guidelines worldwide (34), supporting its inclusion in this review as a common standard in global fracture risk assessment. Among many others groups, the US Preventive Services Task Force (USPSTF) (34), the UK National Osteoporosis Guideline Group (NOGG) (30), the Canadian Task Force on Preventive Health Care (35), and the Groupe de Recherche et d’Information sur les Ostéoporoses (GRIO) in France (36) endorse FRAX for fracture risk assessment.

Several tools recommended by national groups were not included in this review as these tools have been comprehensively evaluated in existing literature. Relatedly, the USPSTF also recommends the Osteoporosis Risk Assessment Instrument (ORAI), Osteoporosis Index of Risk (OSIRIS), Osteoporosis Self-Assessment Tool (OST), and Simple Calculated Osteoporosis Risk Estimation (SCORE), all of which have been reviewed in detail by Wang and colleagues (37). Likewise, Détermination du Risque de Fracture (DeFRA) and Fracture Risk Assessment-Health Statistics (FRA-HS) are recommended by the Italian National Health Institute and have been reviewed in depth by Corrao and colleagues (38). These tools and the relevant literature are summarized in **Tables 1**–**3**.

**Table 2:** 10-year Hip fracture risk prediction tools and discrimination.

Source	Cohort description	Tool and variation	AUC (95% CI where available)^a^	Comparison/Key findings
FRAX development study				
Kanis et al. (2007) (62)	9 prospective population-based cohorts: Rotterdam Study, EVOS/EPOS, CaMos, Rochester, Sheffield, Dubbo, Hiroshima, Gothenburg (2 cohorts) comprising n = 46,340 men and women (68% female, n = 31,511 women, n = 14,829 men)	FRAX with CRFs; no aBMD	0.67 AUC standardized for age 70	*Reference model
Kanis et al. (2007) (62)	9 prospective population-based cohorts; see above	FRAX with CRFs and aBMD	0.78 AUC standardized for age 70	Combining CRFs with aBMD significantly improves risk prediction over using CRFs alone
Additional FRAX literature				
Hippisley-Cox et al., 2009 (68)	UK validation cohort of n = 424,336 men aged 40–85 years	FRAX, no aBMD; Men only	0.817	Reference model
Hippisley-Cox et al., 2009 (68)	UK validation cohort of n = 454,499 women aged 40–85 years	FRAX, no aBMD; Women only	0.845	Reference model
Sambrook et al., 2011 (70)	10 countries (Australia, Belgium, Canada, France, Germany, Italy, the Netherlands, Spain, the UK, and the USA), GLOW cohort of n = 19,586 postmenopausal women aged 60 years or older	FRAX, no aBMD; Women only	0.65	Reference model
Leslie et al., 2010 (69)	Manitoba, Canada, n = 36,730 women (mean age 65.7 +/- 9.8 years) and n = 2,873 men (mean age 68.2 +/- 10.1 years)	FRAX, no aBMD	0.793 (0.775-0.810)	Reference model
Leslie et al., 2010 (69)	Manitoba, Canada, n = 36,730 women (mean age 65.7 +/- 9.8 years) and n = 2,873 men (mean age 68.2 +/- 10.1 years)	FRAX with aBMD	0.830 (0.815-0.846)	Reference model
Leslie et al., 2023 (75)	Manitoba, Canada, n = 71,209 individuals aged 40 years or older (n = 63,917 women, n = 7,292 men, mean age 64.0 +/- 10.7 years)	FRAX with aBMD	0.792 (0.784-0.800)	Reference model
Leslie et al., 2023 (75)	Manitoba, Canada, n = 71,209 individuals aged 40 years or older (n = 63,917 women, n = 7,292 men, mean age 64.0 +/- 10.7 years)	TBS-adjusted FRAX with aBMD	0.794 (0.786-0.802)^c^	Statistically significant difference between TBS-adjusted model and FRAX with aBMD
Leslie et al., 2023 (75)	Manitoba, Canada, n = 71,209 individuals (n = 63,917 women, n = 7,292 men)	FRAX, no aBMD	0.766 (0.756-0.776)	Reference model
Leslie et al., 2023 (75)	Manitoba, Canada, n = 71,209 individuals (n = 63,917 women, n = 7,292 men)	TBS-adjusted FRAX, no aBMD	0.774 (0.764-0.784)^c^	Statistically significant difference between TBS-adjusted model and FRAX without aBMD
Zoulakis et al., 2025 (76)	SUPERB cohort, n = 3,028 older Swedish women aged 75 to 80 years old (mean age 77.8 +/- 1.6 years)	FRAX with aBMD; Women only	0.659 (0.625-0.693)	Reference model
Zoulakis et al., 2025 (76)	SUPERB cohort, n = 3,028 older Swedish women	FRAXplus with aBMD; Women only	0.664 (0.631-0.698)	Reference model
Zoulakis et al., 2025 (76)	SUPERB cohort, n = 3,028 older Swedish women	FRAX, no aBMD; Women only	0.591 (0.554-0.628)	Reference model
Zoulakis et al., 2025 (76)	SUPERB cohort, n = 3,028 older Swedish women	FRAXplus, no aBMD; Women only	0.601 (0.564-0.637)^c^	Statistically significant difference between FRAXplus and FRAX without BMD
Baleanu et al., 2021 (86)	Brussels, Belgium, FRISBEE cohort of n = 3,030 postmenopausal women between ages 60-85	FRAX with aBMD; Women only	0.841 (0.795-0.887)	Reference model
Crandall et al., 2019 (87)	Women’s Health Initiative (WHI) cohort, n = 64,739 postmenopausal American women between ages 50-64 (mean age 57.9 +/- 4.1 years)	FRAX, no aBMD; Women only	0.65 (0.62-0.67)	Reference model
Dagan et al., 2017 (67)	Clalit Health Services (Israel) cohort, n = 1,054,815 Israelis between the ages of 50-90 (n = 478,825 men and n = 575,990 women)	FRAX, no aBMD	0.815 (0.813-0.817)	Reference model
Garvan development study				
Nguyen et al. (2008) (85)	Dubbo study female cohort, n = 1,358 women aged 60 years or older	Garvan Model I with aBMD; Women only	0.757 (standard error, SE: 0.015)	Model I uses age, BMD, prior fractures, and falls history
Nguyen et al. (2008) (85)	Dubbo study male cohort, n = 858 men aged 60 years or older	Garvan Model I with aBMD; Men only	0.754 (SE: 0.024)	N/A
Nguyen et al. (2008) (85)	Dubbo study female cohort, n = 1,358 women aged 60 years or older	Garvan Model II, no aBMD; Women only	0.721 (SE: 0.016)	Model II uses age, weight, prior fractures, and falls history
Nguyen et al. (2008) (85)	Dubbo study male cohort, n = 858 men aged 60 years or older	Garvan Model II, no aBMD; Men only	0.739 (SE: 0.024)	N/A
Additional Garvan literature				
Baleanu et al., 2021 (86)	Brussels, Belgium, FRISBEE cohort of n = 3,030 postmenopausal women between ages 60-85	Garvan with aBMD; Women only	0.769 (0.702-0.836)	Reference model
Crandall et al., 2019 (87)	Women’s Health Initiative (WHI) cohort, n = 64,739 postmenopausal American women aged 50–64 years	Garvan, no aBMD; Women only	0.58 (0.55-0.60)	Reference model
Dagan et al., 2017 (67)	Clalit Health Services (Israel) cohort, n = 1,054,815 Israelis between the ages of 50-90 (n = 478,825 men and n = 575,990 women)	Garvan, no aBMD	0.778 (0.775-0.781)	Reference model
Sambrook et al., 2011 (70)	10 countries (Australia, Belgium, Canada, France, Germany, Italy, the Netherlands, Spain, the UK, and the USA), GLOW cohort of n = 19,586 postmenopausal women aged 60 years or older	Garvan, no aBMD; Women only	0.61	Reference model
QFracture development studies				
Hippisley-Cox et al. (2009) (68)	UK derivation cohort of n = 1,183,663 women aged 30–85 years	QFracture-2009; Women only	0.890 (0.889-0.892)	Reference model
Hippisley-Cox et al. (2009) (68)	UK derivation cohort of n = 1,174,232 men aged 30–85 years	QFracture-2009; Men only	0.856 (0.851-0.860)	Reference model
Hippisley-Cox et al. (2012) (92)	UK derivation cohort of n = 1,598,294 women, aged 30–100 years old	QFracture-2012; Women only	0.893 (0.890-0.896)	Reference model
Hippisley-Cox et al. (2012) (92)	UK derivation cohort of n = 1,544,379 men, aged 30–100 years old	QFracture-2012; Men only	0.875 (0.868-0.883)	Reference model
Additional QFracture literature				
Dagan et al., 2017 (67)	Clalit Health Services (Israel) cohort, n = 1,054,815 Israelis between the ages of 50-90 (n = 478,825 men and n = 575,990 women)	QFracture-2012	0.827 (0.824-0.829)	Reference model
Akyea et al., 2019 (95)	UK primary care COPD cohort, n = 72,586 patients aged 40–90 years	QFracture-2016	0.761 (0.749-0.772)	Reference model, identical performance to FRAX, no aBMD
Akyea et al., 2019 (95)	UK primary care COPD cohort, n = 72,586 patients aged 40–90 years	FRAX, no aBMD	0.761 (0.749-0.772)	Reference model, identical performance to QFracture-2016

The AUC values are included alongside study population descriptions to highlight differences in cohort composition and should not be used to rank tools across studies. Tool comparisons are best interpreted within the same cohort and study context. Calibration is not summarized in this review.

*Reference model indicates that comparisons with other model variations or risk assessment tools were completed within the study.

^a^as reported as recorded in the original publication (95% CI included where available).

^b^Note on FRAX: In the original developmental models (Kanis et al., 2007), “other osteoporotic fractures” (clinical spine, forearm, and proximal humerus) excluded hip fractures, which were modeled as a separate outcome. Subsequent validations use the standard composite Major Osteoporotic Fracture (MOF) definition, which integrates hip fractures alongside spine, forearm, and humerus.

^c^Reported as statistically significant difference between the listed model and the labelled reference model above it.

**Table 3:** 10-year MOF risk prediction tools and discrimination.

Source	Cohort description	Tool and variation	AUC (95% CI where available)^a^	Comparison/Key findings
FRAX development study				
Kanis et al. (2007) (62)	9 prospective population-based cohorts: Rotterdam Study, EVOS/EPOS, CaMos, Rochester, Sheffield, Dubbo, Hiroshima, Gothenburg (2 cohorts) comprising n = 46,340 men and women (68% female, n = 31,511 women, n = 14,829 men)	FRAX with CRFs; no aBMD	0.62^b^ AUC standardized for age 70	*Reference model
Kanis et al. (2007) (62)	9 prospective population-based cohorts; see above	FRAX with CRFs and aBMD	0.63^b^ AUC standardized for age 70	Combining clinical risk factors with femoral neck aBMD did not substantially improve prediction over CRFs alone for other osteoporotic fractures
Additional FRAX literature				
Leslie et al., 2010 (69)	Manitoba, Canada, n = 36,730 women (mean age 65.7 +/- 9.8 years) and n = 2,873 men (mean age 68.2 +/- 10.1 years)	FRAX, no aBMD	0.663 (0.652-0.674)	Reference model
Sambrook et al., 2011 (70)	10 countries (Australia, Belgium, Canada, France, Germany, Italy, the Netherlands, Spain, the UK, and the USA), GLOW cohort of n = 19,586 women aged 60 years or older	FRAX, no aBMD	0.60	Reference model
Leslie et al., 2023 (75)	Manitoba, Canada, n = 71,209 individuals aged 40 years or older (n = 63,917 women, n = 7,292 men, mean age 64.0 +/- 10.7 years)	FRAX with aBMD	0.664 (0.658-0.670)	Reference model
Leslie et al., 2023 (75)	Manitoba, Canada, n = 71,209 individuals (n = 63,917 women, n = 7,292 men)	TBS-adjusted FRAX with aBMD	0.673 (0.667-0.679)^c^	Statistically significant difference between TBS-adjusted model and FRAX with aBMD
Leslie et al., 2023 (75)	Manitoba, Canada, n = 71,209 individuals (n = 63,917 women, n = 7,292 men)	FRAX, no aBMD	0.639 (0.631-0.647)	Reference model
Leslie et al., 2023 (75)	Manitoba, Canada, n = 71,209 individuals (n = 63,917 women, n = 7,292 men)	TBS-adjusted FRAX, no aBMD	0.654 (0.646-0.662)^c^	Statistically significant difference between TBS-adjusted model and FRAX without aBMD
Zoulakis et al., 2025 (76) (Supplementary information)	SUPERB cohort, n = 3,028 Swedish women aged 75 to 80 years old (mean age 77.8 +/- 1.6 years)	FRAX with aBMD	0.625 (0.603-0.647)	Reference model
Zoulakis et al., 2025 (76)	SUPERB cohort, n = 3,028 Swedish women	FRAXplus with aBMD	0.631 (0.609-0.653)^c^	Statistically significant difference between FRAXplus and FRAX with aBMD
Zoulakis et al., 2025 (76)	SUPERB cohort, n = 3,028 Swedish women	FRAX, no aBMD	0.570 (0.547-0.593)	Reference model
Zoulakis et al., 2025 (76)	SUPERB cohort, n = 3,028 Swedish women	FRAXplus, no aBMD	0.577 (0.554-0.600)^c^	Statistically significant difference between FRAXplus and FRAX without aBMD
Dagan et al., 2017 (67)	Clalit Health Services (Israel) cohort, n = 1,054,815 Israelis between the ages of 50-90 (n = 478,825 men and n = 575,990 women)	FRAX, no aBMD	0.714 (0.712-0.716)	Reference model
QFracture development studies				
Hippisley-Cox et al. (2009) (68)	UK derivation cohort of n = 1,183,663 women aged 30–85 years	QFracture-2009; Women only	0.79 (0.786-0.790)	Reference model
Hippisley-Cox et al. (2009) (68)	UK derivation cohort of n = 1,174,232 men aged 30–85 years	QFracture-2009; Men only	0.69 (0.684-0.692)	Reference model
Hippisley-Cox et al. (2012) (92)	UK derivation cohort of n = 3,142,673 patients aged 30–100 years (n = 1,598,294 women, n = 1,544,379 men)	QFracture-2012; Women only	0.79 (0.787-0.793)	Reference model
Hippisley-Cox et al. (2012) (92)	UK derivation cohort of n = 3,142,673 patients aged 30–100 years (n = 1,598,294 women, n = 1,544,379 men)	QFracture-2012; Men only	0.71 (0.703-0.719)	Reference model
Additional QFracture literature				
Dagan et al., 2017 (67)	Clalit Health Services (Israel) cohort, n = 1,054,815 Israelis between the ages of 50-90 (n = 478,825 men and n = 575,990 women)	QFracture-2012	0.712 (0.710-0.714)	Reference model
Akyea et al., 2019 (95)	UK primary care COPD cohort, n = 72,586 patients aged 40–90 years	QFracture-2016	0.614 (0.605-0.623)	Reference model
Akyea et al., 2019 (95)	UK primary care COPD cohort, n = 72,586 patients aged 40–90 years	FRAX, no aBMD	0.714 (0.706-0.722)	FRAX discrimination of MOF was greater compared to QFracture
µFRAC development study				
Whittier et al. (2023) (99)	BoMIC cohort, n = 6,802 participants (n = 4,829 women, n = 1,973 men, mean age 70.1 +/- 8.0 years)	µFRAC (complete model)	0.733 (0.729-0.738)	Full model uses standard HR-pQCT parameters, FEA failure load, CRFs, and FN aBMD
Whittier et al. (2023) (99)	BoMIC cohort, n = 6,802 participants (n = 4,829 women, n = 1,973 men)	µFRAC’ (simplified model)	0.722 (0.718-0.726)	Simpler model uses age, standard HR-pQCT microarchitecture parameters, and FEA failure load
Whittier et al. (2023) (99)	BoMIC cohort, n = 6,802 participants (n = 4,829 women, n = 1,973 men)	µFRAC’’ (simplest model)	0.715 (0.711-0.719)	Simplest model considers age and standard HR-pQCT microarchitecture parameters
Additional µFRAC literature				
Bugbird et al., 2026 (113)	MrOS cohort, n = 1,586 community-dwelling American men aged 77 or older (mean age 84.4 +/- 4.2 years)	µFRAC (complete model)	0.687 (0.682-0.692)	Reference model
Bugbird et al., 2026 (113)	MrOS cohort, n = 1,586 community-dwelling American men	FRAX with aBMD	0.613 (0.609-0.617)^c^	Statistically significant lower AUC compared to complete µFRAC model
Bugbird et al., 2025 (39)	SUPERB cohort, n = 2,307 Swedish women aged 75 to 80 years old (average age 77.7 +/- 1.6 years)	µFRAC (complete model)	0.637 (0.634-0.640)	Reference Model
Bugbird et al., 2025 (39)	SUPERB cohort, n = 2,307 Swedish women	FRAX with aBMD	0.628 (0.625-0.632)^c^	Statistically significant lower AUC compared to complete µFRAC model

The AUC values are included alongside study population descriptions to highlight differences in cohort composition and should not be used to rank tools across studies. Tool comparisons are best interpreted within the same cohort and study context. Calibration is not summarized in this review.

*Reference model indicates that comparisons with other model variations or risk assessment tools were completed within the study.

^a^as reported as recorded in the original publication (95% CI included where available).

^b^Note on FRAX: In the original developmental models (Kanis et al., 2007), “other osteoporotic fractures” (clinical spine, forearm, and proximal humerus) excluded hip fractures, which were modeled as a separate outcome. Subsequent validations use the standard composite Major Osteoporotic Fracture (MOF) definition, which integrates hip fractures alongside spine, forearm, and humerus.

^c^Reported as statistically significant difference between the listed model and the labelled reference model above it.

The studies included in **Tables 2**, **3**, section “[model name] Development Study”, represent the study/studies undertaken to develop the model (i.e., the primary purpose of the study was to develop the fracture risk prediction tool). These studies were identified by reviewing the reference lists of each of the clinical guidelines listed above. The studies included in the “Additional [model name] literature” section represent selected examples from the literature to illustrate validation studies for the various tools. These studies do not represent a comprehensive systematic review of validation studies for the included tools.

Important characteristics to consider when evaluating fracture risk assessment tools are discrimination (the degree to which the model differentiates those at higher risk of having a fracture from those at lower risk) and calibration (how similar the predicted absolute risk of fracture is to the true, or also referred to as observed, risk of fracture in groups of patients classified in different risk strata). For the purposes of this review, we focus on the Area Under the Receiver Operating Characteristic Curve (AUC-ROC) as a measure of discrimination. We report the AUC value and associated 95% confidence interval as reported in the original publications. Where available, we include descriptive information on the cohort the tools were evaluated on. Generally, AUC scores above 0.9 are deemed to have excellent discrimination (39) (i.e. the probability of an individual with a fracture having a higher fracture risk assessment score than an individual without a fracture is 90%). Scores between 0.8-0.9 are considered good, where greater than 0.8 typically means a tool has clinical utility. Between 0.7-0.8 indicates acceptable performance, and between 0.6-0.7 is sub-optimal discrimination. Scores between 0.5-0.6 are considered only slightly better than chance (i.e. poor discrimination). AUC is not an exhaustive metric to evaluate all elements of model performance, but its inclusion in this review is to provide an overview of current predictive performance rather than providing a broad tool hierarchy. AUC is influenced by cohort characteristics such as age, baseline fracture risk, follow-up duration, and outcome definitions. AUC serves as one important metric to compare fracture risk assessment tools, however calibration, net reclassification [the degree to which a new fracture risk assessment tool reclassifies individuals into various risk categories compared with a previous tool (40)] and clinical integration are essential features to consider when comparing fracture risk assessment tools. Though calibration is crucial for assessing the accuracy of predicted risk, this review focuses on discrimination across tools and does not summarize calibration.

### 1.2 DXA as a clinical standard

As an imaging modality, DXA is a clinical standard for fracture risk assessment and offers broad availability and integration into clinical decision-making frameworks. An advantage of DXA is it outputs standardized comparisons of aBMD, which supports integration and adoption within clinical practice. When referencing aBMD in this review, we specifically refer to DXA-derived aBMD and not aBMD from other modalities (e.g. Radiofrequency Echographic Multi-Spectrometry). Although DXA was introduced for clinical use in the late 1980s (41, 42), early clinical reference databases were proprietary and specific to scanner vendors. Standardized fracture risk assessment using a normative reference database did not become widely accessible until population-based datasets like the National Health and Nutrition Examination Survey (NHANES) were made publicly available, which allowed for BMD comparisons at the femoral neck across different DXA vendors (41, 43). In 2005, the International Society for Clinical Densitometry (ISCD) approved the NHANES dataset as the official standardized reference dataset for hip scores (44). Relevantly, comparative aBMD scores at the femoral neck (FN.aBMD) are used to diagnose and assess osteoporosis and fracture risk. An operational definition of osteoporosis is provided by the World Health Organization, which is an aBMD below 2.5 standard deviations or greater below the average mean value of a young, healthy reference population from the NHANES III dataset (T-score ≤ −2.5). A key strength of DXA is its ability to contextualize bone density against a reference population, allowing for standardized risk stratification for individuals. The developers of the DXA reference standards have encouraged that advancements in fracture prediction coincide with reassessments of existing reference standards (45). Accordingly, fracture risk prediction technologies are underpinned by comparisons with population-based reference datasets.

Though DXA is favorable for clinical integration and its utility is supported by interpretability of reference data, it cannot directly measure true volumetric density. Because aBMD is calculated as an integral of bone mineral content over a given two-dimensional projection, aBMD is influenced by bone size (46, 47). Two individuals with comparable volumetric bone mineral density (vBMD) but different bone sizes will differ in aBMD and therefore in their DXA-based fracture risk assessment. Consequently, aBMD may overestimate fracture risk in individuals with smaller skeletal size and underestimate risk in individuals with larger skeletal size, independent of true bone properties (47). Due to smaller bone size, Asian women often have lower aBMD values compared with Caucasian women, yet experience significantly lower hip fracture rates (48). This discrepancy between aBMD and observed fracture incidence in Asian women may be attributed to protective features like favorable hip geometry, cortical thickness, and trabecular BMD, demonstrating that aBMD may not stratify fracture risk appropriately across all groups (48). These limitations highlight the need for bone imaging capable of directly characterizing bone density in three-dimensions, to capture aspects of bone quality beyond aBMD.

Notably, in the Rotterdam Study, 66% of non-vertebral fractures in women and 79% in men occurred in individuals with non-osteoporotic BMD (49). This discrepancy of DXA in identifying individuals at risk is further emphasized when considering DXA aBMD AUC values for osteoporotic fractures range from suboptimal (AUC = 0.59-0.61) (50, 51) to acceptable (AUC = 0.732) (52). Moreover, aBMD cannot differentiate between bone compartments (i.e. trabecular and cortical compartments), meaning it is not possible to track compartment-specific features that contribute to fracture risk (53). Attempts to address this limitation of DXA include the Trabecular Bone Score (TBS), which quantifies trabecular bone using two-dimensional, gray-level textural analysis of DXA spine images (54, 55).

While HR-pQCT directly measures bone microarchitecture, TBS provides an estimate of spine microarchitecture that has been associated with both trabecular and cortical bone parameters measured by HR-pQCT (54, 55). However, the strength of these associations varies, with some studies finding very weak to moderate associations between TBS and HR-pQCT-derived vBMD and microarchitectural measures (54–57). Moreover, these relationships differ by trabecular and cortical parameters (56, 57). TBS may be reflective of general skeletal health (54, 55) and provide additional information for fracture risk prediction (28). Though TBS cannot directly predict fracture risk, it is integrated as a CRF in existing fracture prediction tools. Relevantly, fracture risk tools integrate aBMD with other CRFs like TBS to overcome the limitations of using aBMD alone for fracture discrimination.

Despite being an indirect measure of bone, aBMD is a reasonable surrogate for bone strength (58, 59), and is often used in fracture prediction tools. This represents a key challenge in current fracture risk assessment because the primary measure of bone is inherently restricted as an estimate (60). For this reason, fracture risk tools are strongly supplemented by CRFs (61). In fact, in many fracture prediction tools aBMD is simply considered a CRF despite the obvious central importance of bone quality on fractures. In the following sections, commonly used fracture risk assessment tools are described.

### 1.3 FRAX

FRAX is a fracture risk assessment tool that incorporates CRFs and, if available, aBMD values of the femoral neck (62, 63). First released in 2008, FRAX is now the most widely used fracture risk assessment tool (64). FRAX was initially developed using nine population-based cohorts and validated in 11 independent cohorts spanning North America, Europe, Asia, and Australia (62, 63, 65). More recent efforts have focused on updating the predictive value of FRAX clinical risk factors, such as BMD, using data from 64 international cohorts (n=2,138,428 men and women) (24). FRAX outputs the 10-year risk of a hip fracture and the 10-year risk of a major osteoporotic fracture (MOF; includes the hip, spine, forearm, or humerus). FRAX incorporates the following 10 CRFs and can be calculated with and without aBMD: age, sex, body mass index (BMI), previous fracture, family history of hip fracture, smoking status, glucocorticoid use, diagnosis of rheumatoid arthritis, secondary osteoporosis and alcohol intake (**Table 1**). Secondary osteoporosis conditions included in FRAX are type 1 diabetes, osteogenesis imperfecta in adults, hypogonadism, menopause before the age of 45, chronic malnutrition, chronic malabsorption, chronic renal failure, chronic liver disease, untreated long-term hyperthyroidism, and osteogenesis imperfecta in adults. FRAX is calibrated for country-specific populations, with regional fracture incidence rates integrated in models specific to over 80 countries including Canada, the United States, and the United Kingdom.

Several features of FRAX support its clinical practicality. The utility of FRAX is strengthened by the fact that 10-year fracture risk can be calculated without aBMD. This makes FRAX possible to be used as a screening tool without the requirement of using scarce imaging resources (e.g. physician’s office, DXA imaging). Some studies have found that FRAX without aBMD can achieve comparable fracture risk performance to aBMD (66), though both are limited in their fracture risk discrimination abilities. Additionally, FRAX considers the competing risk of mortality in its fracture risk score for the assessment of absolute 10-year fracture probability in older populations (64). By considering population-specific life expectancy, the FRAX approach to modeling accounts for the possibility that an individual may pass away before a potential fracture occurs. This explains why FRAX’s predicted-to-observed ratios for fracture risk remains relatively consistent with aging compared to other prediction tools (67).

The performance of FRAX with and without aBMD has been assessed in large cohorts, revealing suboptimal-to-acceptable discrimination of hip fractures, and suboptimal discrimination of MOFs (**Tables 2** and **3**). FRAX 10-year risk of hip fracture without aBMD demonstrated strong discrimination of hip fractures in a United Kingdom (UK) cohort of 424,336 men and 454,499 women aged 40-85, with AUC values of 0.845 in women and 0.817 in men (68). A Canadian study using the Manitoba Bone Density Program database found similar results for FRAX 10-year risk of hip fracture without aBMD in a cohort of 36,730 women (mean age 65.7 ± 9.8) and 2,873 men (mean age 68.2 ± 10.1), with an overall AUC of 0.793 (95% CI: 0.775-0.810) (69). When aBMD was included in FRAX (i.e. 10-year risk of hip fracture with aBMD), the Canadian study noted improvements in hip fracture discrimination (0.830, 95% CI: 0.815-0.846) (69). Conversely, the international GLOW study of 19,586 women aged 60 years or older across 10 countries (Australia, Belgium, Canada, France, Germany, Italy, the Netherlands, Spain, the UK, and the USA) reported lower hip fracture discrimination with an AUC of 0.65 using FRAX 10-year risk of hip fracture without aBMD (70). In all three studies (UK, Canadian, and GLOW), the ability of FRAX to discriminate was stronger for hip fractures than for MOF. Using FRAX 10-year risk of MOF without aBMD, the Canadian study found that MOF fracture discrimination was suboptimal (0.663, 95% CI: 0.652-0.674) (69), while the international GLOW study found that MOF discrimination was limited (0.60) (70). Though FRAX demonstrates greater discrimination for hip fractures than MOFs, further refinement is needed to improve performance across all cohorts and outcomes.

Although FRAX is the most prevalent fracture risk assessment tool, there are several limitations. Comparative analyses with other fracture risk assessment tools demonstrate that other tools may outperform FRAX in identifying individuals who later fracture (67), albeit with conflicting results. An additional limitation of FRAX is that the tool relies on calibration using country-specific epidemiological data (71). However, a FRAX model is not available for every country because some regions may lack the fracture incidence data required for model development, although there has been significant progress regarding the number of included countries. An approach that incorporates the direct assessment of bone, which we will discuss later (e.g., HR-pQCT), may reduce the reliance on epidemiological data, as risk estimation would be established using patient-specific measures of bone rather than relying on calibration to country-level fracture incidence and mortality rates. Like many tools, FRAX does not account for history of falls or other emerging contributors to fracture risk, like fall-risk-increasing drugs (FRIDs), muscle strength and function, or physical performance measures, which are increasingly considered potential drivers of fracture risk by identifying individuals who are at increased risk for falls. Moreover, FRAX incorporates a history of clinical fractures as a binary variable (72), which prevents users from inputting multiple fragility fractures. As a dose-dependent relationship has been established with the number of prior fragility fractures, FRAX may underestimate risk in individuals with multiple fractures (73, 74). While many of these limitations might be addressed in the future, the advent of FRAXplus is already addressing some of these.

Relevantly, the inclusion of TBS in FRAXplus allows a surrogate measure of microarchitecture to be considered when calculating fracture risk scores. However, existing work directly evaluating the influence of TBS on fracture prediction has assessed TBS-adjusted conventional FRAX rather than FRAXplus (75). Using the Manitoba BMD Registry of 71,209 individuals aged 40 years or older (89.8% female; mean age 64.0 ± 10.7 years), Leslie and colleagues evaluated how TBS influenced fracture prediction when incorporated into FRAX. A significant, modest increase in AUC of 0.009 was noted for MOF prediction when FRAX with BMD was compared to TBS-adjusted FRAX with BMD, with AUC rising from 0.664 (95% CI: 0.658-0.670) to 0.673 (95% CI: 0.667-0.679). When BMD was not used in FRAX, the TBS adjustment also demonstrated a significant, modest improvement in discrimination of MOFs, with AUC increasing from 0.639 (95% CI: 0.631-0.647) to 0.654 (95% CI: 0.646-0.662). Similar improvements were observed for hip fracture prediction (**Table 1**) (75). Leslie and colleagues’ findings support a modest benefit of incorporating a microarchitecture surrogate into FRAX-based fracture risk assessment, however, whether this improvement translates directly to FRAXplus remains unclear.

#### 1.3.1 FRAXplus

FRAXplus is an extended version of the FRAX prediction tool. As opposed to FRAX which is freely available for unlimited calculations, FRAXplus uses a tiered subscription model. A free FRAXplus account allows up to five fracture assessments, whereas paid plans allow an unlimited number of assessments. In addition to including the CRFs included in the original FRAX model, FRAXplus also considers adjustments for recency and site of osteoporotic fractures, dosage of oral glucocorticoids, falls in the last year, hip axis length, duration of type 2 diabetes mellitus, and provides optional inputs for lumbar spine TBS and FN.aBMD (76). While FRAX is intended for general screening to identify individuals at risk of fragility fracture, FRAXplus is developed for personalized fracture risk assessment (76). By accounting for risk factors not included in the FRAX model, FRAXplus may improve risk stratification by identifying individuals whose fracture risk may be underestimated by the FRAX. These cases may appear low risk with FRAX but are found to have a significantly increased risk of fracture with the inclusion of additional risk factors incorporated in FRAXplus (76).

There is currently limited evidence comparing the performance of FRAX and FRAXplus. However, one study on the Sahlgrenska University Hospital Prospective Evaluation of Risk of Bone Fractures (SUPERB) cohort provides some insight. In the SUPERB cohort of 3,028 older Swedish women aged 75 to 80 years old (76), the inclusion of additional CRFs in FRAXplus increased the percentage of women above interventional thresholds for osteoporosis treatment to 41.1%, compared with 29.9% using conventional FRAX. Without aBMD, FRAXplus compared to FRAX for 10-year risk of hip fracture demonstrated a small but statistically significant improvement in hip fracture discrimination (p = 0.025), with AUC scores of 0.601 (95% CI: 0.564-0.637) and 0.591 (95% CI: 0.554-0.628), respectively. When aBMD inputs were included in the models, no statistically significant improvement was observed between the FRAX and FRAXplus for 10-year risk of hip fracture (**Table 2**). However, while FRAX 10-year risk of MOF with aBMD produced an AUC of 0.625 (95% CI: 0.603–0.647), FRAXplus 10-year risk of MOF with aBMD showed a small improvement with an AUC of 0.631 (95% CI: 0.609–0.653, p = 0.038). FRAX 10-year risk of MOF without aBMD had an AUC of 0.570 (95% CI: 0.547–0.593) while FRAXplus had an AUC of 0.577 (95% CI: 0.554–0.600) (**Table 3**) (76). The changes in MOF AUCs between FRAX and FRAXplus with and without BMD were statistically significant, indicating that FRAXplus may improve MOF discrimination. However, even with the inclusion of additional CRFs, FRAXplus discrimination appears limited and the improvements are modest.

### 1.4 CAROC

CAROC was developed by the Canadian Association of Radiologists and Osteoporosis Canada introduced in 2005 (77). The CAROC model inputs include FN.aBMD, age, sex, fragility fracture history after 40, and glucocorticoid use (**Table 1**). CAROC reports risk using a semi-quantitative model, with 10-year risk of MOF categorized as low (<10%), moderate (10–20%), or high (>20%). As individuals are categorized into low, moderate or high risk, AUC for CAROC is typically not assessed. Instead, CAROC’s performance is often evaluated using agreement with other tools like FRAX.

A benefit of CAROC is that its parsimonious design makes it suitable for time-limited environments (78). CAROC can inform interventional approaches without requiring a detailed patient history like FRAX or QFracture (discussed below). CAROC has been described as transparent in its risk categorization approach, with the risk determination methodology publicly available and interpretable in tables for clinicians to verify when assessing a patient’s risk (72).

Despite its simple design, CAROC can accurately categorize individuals into fracture risk categories. In a clinical cohort of 36,730 women and 2,873 men (mean age 65.7 ± 9.8 and 68.2 ± 10, respectively), CAROC stratified men and women into risk categories that aligned with observed fracture incidence rates, indicating it was adequately calibrated (79). A significant predictive gradient (p < 0.001) was found in both men and women, with observed 10-year fracture rates increasing progressively across the low, moderate, and high-risk categories. The CAROC system also demonstrated agreement with FRAX in two Canadian cohorts, with 88-89% concordance in risk categorization (80); here, it was observed that the tools provided comparable categorical risk stratification at the population level. However, discordance of fracture risk prediction between FRAX and CAROC was present for some edge cases. A later direct comparison of 34,060 individuals (mean age 66.6 years, 91.0% women) found that FRAX provided a modest but statistically significant improvement in risk stratification over CAROC (81).

Unlike FRAX, which has an option to estimate 10-year risk of fracture using CRFs alone, CAROC requires DXA-derived aBMD (82). Additionally, it has been demonstrated in a randomized controlled trial that clinicians prefer FRAX-based reporting compared to CAROC reports, and that clinicians complete more thorough follow-up for patients who receive FRAX reports compared to those who receive CAROC reports (83). Comparative analyses have further indicated that CAROC tends to overestimate 10-year fracture risk, such as classifying a patient as high risk when their actual observed outcomes align more closely with a moderate category (79, 81). Finally, CAROC does not consider fall risk in its modeling despite its identification as an independent contributor to fracture risk (84). Despite these limitations, the 2023 Osteoporosis Canada guidelines consider CAROC as a suitable alternative fracture risk assessment tool, though the Canadian FRAX is recommended as the preferred tool (3).

### 1.5 Garvan fracture risk calculator

The Garvan Fracture Risk Calculator (Garvan) was developed and released in 2008 by the Garvan Institute of Medical Research in Australia (85). It was developed using the Dubbo Osteoporosis Epidemiology Study, a cohort of 2,216 individuals (61.3% female) aged 60 years and older (85). Garvan inputs are age, sex, weight, fracture history after 50, fall history (recorded as 0, 1, 2, or 3+ in the last 12 months), and optionally, aBMD (**Table 1**). Body weight is only used in the Garvan tool if aBMD is not provided. Garvan outputs 5- and 10-year risk of hip fracture and ‘any osteoporotic fracture’. Garvan’s ‘any osteoporotic fracture’ category is commonly referred to as ‘any Garvan fracture’ and includes fractures of the hip, wrist, metacarpals, humerus, scapula, clavicle, proximal tibia, pelvis, sternum, distal femur, and patella, and symptomatic vertebral fractures.

Of the existing fracture risk assessment tools, Garvan is distinct in its integration of falls as a dose-dependent variable. The tool provides 5- and 10-year risk of hip fracture and ‘any fragility fracture’ that accounts for the rising cumulative risk associated with repeated fall events (85). Moreover, other fracture risk assessment tools in this review focus on hip fractures and MOFs (defined as hip, clinical spine, humerus, and wrist fractures). By broadening fracture risk assessment to include ‘any Garvan fracture’, Garvan can help identify individuals whose quality of life may still be impacted by fragility fractures, even if they do not experience the most clinically severe fracture types. Garvan is also convenient for clinical use because imaging is not required to use the tool, reducing its reliance on imaging resources. Like CAROC, Garvan has a simplified design (85). The information required to complete a Garvan assessment is simple compared to other fracture risk tools and can be collected during routine clinical workups. Consequently, Garvan is suitable for resource-limited and time-limited settings.

Garvan’s performance varies significantly across demographics. Performance between Garvan and FRAX was compared using the Fracture Risk Brussels Epidemiological Enquiry (FRISBEE) dataset in 3,030 postmenopausal women between ages 60–85 with 5 years of follow-up (86). For 10-year hip fracture prediction, FRAX with aBMD outperformed Garvan’s 10-year risk of hip fracture with aBMD (AUC = 0.841, 95% CI: 0.795-0.887, and AUC = 0.769, 95% CI 0.702-0.836, respectively) (**Table 2**). Moreover, though Garvan was developed using data from individuals aged 65 or older, it is also used to assess fracture risk in individuals aged 50–96 years old. Garvan’s hip fracture discrimination in 64,739 postmenopausal American women between the ages of 50–64 demonstrated suboptimal performance. Within this cohort and without aBMD, the Garvan 10-year risk of hip fracture had an AUC of 0.58 (95% CI: 0.55-0.60) compared to the 0.65 (95% CI 0.62-0.67) AUC of FRAX (87). In a small Australian cohort of 200 men and women aged 60–90 years (72% women), Garvan with aBMD was evaluated for detecting 10-year ‘any Garvan fracture’ while FRAX with aBMD was evaluated for detecting 10-year MOF (88). Direct AUC comparison in this study is challenged because Garvan and FRAX were evaluated for different outcomes, but this work is included to instead highlight sex-based differences in tool performance. Among women, Garvan achieved an AUC of 0.84 for 10-year ‘any Garvan fracture’ while FRAX achieved AUCs of 0.77-0.78 for 10-year MOF depending on the FRAX version used. Among men, Garvan achieved an AUC of 0.76 for 10-year ‘any Garvan fracture’ while the FRAX versions had AUCs of 0.54 and 0.57 for 10-year MOF (88).

A limitation of Garvan is that although it accounts for fall risk, it does not consider other significant CRFs that could be obtained during routine clinical encounters. For instance, smoking status, alcohol intake, and glucocorticoid use are known independent risk factors of fragility fractures (89, 90) yet are not considered in Garvan. An additional limitation of Garvan is that the observed-to-predicted ratios of fracture decreases as age increases (67). Comparatively, FRAX’s observed-to-predicted ratio appears more consistent with age, which may be due to its consideration of the competing risk of death. Finally, Garvan was developed using a database of primarily Caucasian individuals, with 98.6% of the cohort identified as Caucasian (85). This may limit its performance in certain groups, such as African American women (87), although this limitation is not necessarily unique to Garvan.

### 1.6 QFracture

QFracture was developed at the University of Nottingham and released in 2009 (68). The tool provides a 10-year risk of osteoporotic fracture (defined as fracture of the hip, wrist, shoulder, or spine) and hip fracture, and has been independently validated (91). QFracture once defined ‘osteoporotic fracture’ as a fracture of the wrist, spine, or hip (QFracture2009), but this outcome definition has been expanded to now include shoulder fractures in more recent models (QFracture-2012, QFracture-2016) (92). The updated QFracture definition of ‘osteoporotic fracture’ now aligns with the MOF definition used in FRAX, FRAXplus, and CAROC; accordingly, the discriminative performance of QFracture-2012 and QFracture-2016 is compared to both MOFs and hip fractures in this review. QFracture-2012 is discussed in greater detail in this review (**Tables 2** and **3**) as it is the version most frequently evaluated in existing comparative studies. However, developer-reported validation suggests that QFracture-2016 has improved osteoporotic fracture discrimination compared to QFracture-2012 (93).

The QFracture calculator can be adjusted to produce a cumulative absolute risk prediction over a customizable 1- to 10-year time frame. In addition to updating the fracture outcome definition in QFracture-2012, the included CRFs were also expanded (92). QFracture CRFs include age, sex, BMI (optional), ethnicity, fall history, diabetes, cardiovascular disease, use of hormone replacement therapy, tricyclic antidepressants, asthma, smoking status, alcohol consumption, parental history of hip fracture or osteoporosis, rheumatoid arthritis or systemic lupus erythematosus, chronic liver disease, gastrointestinal malabsorption, corticosteroids, endocrine conditions, dementia, Parkinson’s disease, epilepsy, cancer history, chronic renal disease, and nursing or care home residence (**Table 1**). QFracture does not include aBMD and has no option to consider it in the risk calculation.

A strength of QFracture is its reliance on 26 variables that are available in electronic health records (EHR). This reliance on EHR is intentional as QFracture was designed to minimize use of additional clinical resources, and instead to rely on existing medical records (92). QFracture can also provide fracture risk scores at 1-10-year intervals, supporting the interpretation of both immediate and longer-term fracture risk. QFracture is advantageous because it can be applied across a broader age range (30–100 years) than many other fracture risk assessment tools. In comparative analyses, QFracture has demonstrated comparable and superior performance to FRAX and Garvan models that incorporate aBMD (67), despite not including aBMD. Moreover, though QFracture was developed in the UK, there are no CRFs in the model that are UK-specific (91), which makes it possible to adapt QFracture for use in populations outside of the UK (91), but, this would require validation. The integration of dose-response relationships for smoking and alcohol also supports the model’s performance, as fracture risk varies depending on the amount of exposure to CRFs. Using AUC, QFracture has demonstrated acceptable-to-excellent discriminatory performance (94).

QFracture has been evaluated on large cohorts, revealing acceptable-to-good fracture discrimination across cohorts and fracture outcomes. QFracture-2012 was internally validated in a large UK primary care cohort of 1,583,373 patients (50.8% women) using prospective data, demonstrating excellent discrimination of hip fractures with AUC of 0.893 in women (95% CI: 0.890-0.896) and 0.875 in men (95% CI: 0.868-0.883) (92). In a retrospective study with 1,054,815 Israelis between the ages of 50-90 (54.6% women), the performance of QFracture-2012, FRAX without BMD, and Garvan without BMD were compared (67) (**Tables 2** and **3**). For hip fractures, QFracture-2012 produced an AUC of 0.827 (95% CI: 0.824-0.829), demonstrating stronger discriminatory abilities in this study compared to FRAX and Garvan, which had AUCs of 0.815 (95% CI: 0.813-0.817) and 0.778 (95% CI: 0.775-0.781), respectively (67). The same study found that discrimination for QFracture and FRAX were similar. For MOF, QFracture-2012 demonstrated an AUC of 0.712 (95% CI: 0.710-0.714) and FRAX with an AUC of 0.714 (95% CI: 0.712-0.716) (67). The discrimination of QFracture-2016 and FRAX has also been assessed on chronic obstructive pulmonary disease (COPD) populations, revealing FRAX without BMD outperformed QFracture for MOF discrimination (0.714, 95% CI: 0.706-0.722 and 0.614, 95% CI: 0.605-0.623 respectively). However, both models had identical and acceptable performance in hip fracture discrimination in the COPD population (0.761, 95% CI: 0.749-0.772) (95). Overall, comparisons across large cohorts demonstrate strong QFracture discrimination for hip fracture but more variable performance for MOFs.

Despite the fairly consistent and acceptable-to-good fracture discrimination of QFracture, it has several limitations. Though QFracture relies on EHR information, the substantial number of inputs is a disadvantage for routine implementation. A criticism of QFracture, as well as Garvan, is that the tools do not consider the competing risk of mortality in their assessments (67); this may contribute to the overestimations of fracture risk seen in older individuals (67, 96). Relatedly, QFracture may underestimate fracture risk in younger people (96). Another limitation of QFracture is that for certain inputs like fall history with known dose-dependent responses, the tool restricts input to binary yes/no values. Finally, the inclusion of a broader age range in QFracture compared to other fracture risk tools may overstate its reported AUC performance (67). A broader age range means the model must differentiate risk between low-risk young individuals and relatively higher-risk older individuals. As age is a strong determinant of fracture risk, adjustment for age likely contributes substantially to the model’s AUC performance.

### 1.7 Meta-regression models and comparative performance of current prediction tools

As highlighted throughout this review, current fracture risk prediction tools have important advantages and limitations. Consequently, determining whether any existing tool is superior for fracture risk prediction remains challenging. Meta-analyses and reviews comparing the discriminative performance of existing fracture risk prediction tools aim to address this question (28, 97, 98). Beaudoin and colleagues assessed fracture discrimination abilities across 14 externally validated tools, producing unadjusted AUC comparisons. Of the 14 tools evaluated, FRAX, Garvan, and QFracture were further assessed using meta-regression models that controlled for sex, mean age, age range, and study quality, to produce adjusted AUC comparisons. Importantly, Beaudoin and colleagues emphasized that comparisons between fracture prediction tools are limited by the heterogeneity across studies. This is echoed in the findings of Marques and colleagues, who also identified study heterogeneity as a challenge when comparing fracture prediction tools in their earlier meta-analysis (97).

Accordingly, meta-regression models are used to increase comparability between prediction tool studies by accounting for the influence that study characteristics have on performance comparisons. For instance, in the adjusted AUC comparison FRAX with BMD had the greatest discrimination for hip fracture (AUC = 0.81, 95% CI: 0.78-0.84), while in the unadjusted analyses, QFracture2009 had the greatest hip fracture discrimination (AUC = 0.88, 95% CI: 0.86-0.89). Evidently, adjusting for differences in baseline cohort characteristics can influence model performance when comparing the discrimination of fracture risk prediction tools.

## 2 Emerging tools in fracture risk prediction

The inclusion of CRFs to predict osteoporotic fracture are central to the identification of individuals at risk of fracturing using fracture risk assessment tools. However, as noted, most fracture risk assessment tools use indirect measures of bone strength such as aBMD, or approximate bone quality based on anthropometric measures like BMI. Conversely, there are emerging imaging tools such as HR-pQCT that can integrate volumetric information on bone density and structure into prediction models (99). As HR-pQCT is a relatively new form of densitometry compared to DXA, and although generally used for research rather than clinical assessment, the development of fracture risk assessment tools that integrate HR-pQCT measures of BMD and microarchitecture features is ongoing and expanding. Although HR-pQCT-based approaches have been proposed, no fracture risk assessment tool has emerged as an established standard. We will examine the current status of this technology and propose future directions.

HR-pQCT is a low-dose radiation imaging modality that outputs 3D information on bone microarchitecture of cortical and trabecular compartments at peripheral sites (e.g. distal radius and tibia) (100). HR-pQCT provides comprehensive information on structural features of bone compartments, allowing the exploration of how these features contribute to skeletal frailty. BMD and microarchitecture are collected in resolutions at the micrometer scale, with first-generation HR-pQCT (XtremeCT) and second-generation HR-pQCT (XtremeCT II) offering nominal resolutions of 82 µm and 60.7 µm isotropic voxel size, respectively (100). HR-pQCT outputs volumetric bone mineral density (vBMD) that is used as a surrogate measure of bone strength, but unlike DXA-dervied aBMD, it is a measure of bone mineral content per unit volume (g/cm^3^), not area. HR-pQCT-based fracture prediction models typically combine vBMD and bone microarchitecture outputs of both cortical and trabecular compartments in their algorithms.

Since its release in 2005, one of the main challenges of HR-pQCT is the interpretation of the complex data. Much like the NHANES III reference dataset for DXA with T- and Z-scores, several groups have provided HR-pQCT reference sets with T- and Z-scores. This is especially useful when considering the high-dimensional data output by HR-pQCT scanners, which can be challenging to interpret and assess individual bone health. HR-pQCT normative datasets with age, sex, and site-specific microarchitecture and vBMD centiles have been developed to allow age-matched adults and adolescents (101, 102). Our lab built an online resource called Normative (https://normative.ca) that provides access to these centile reference curves, improving the accessibility and interpretation. Similarly, Warden and colleagues have curated a reference dataset and calculator (103). While these are important steps toward standardization, no single reference standard has yet to be universally adopted. Consideration for combining reference data or building new reference data may be warranted. Nevertheless, while reference data is important for the interpretation of HR-pQCT data, it is not a substitute for fracture risk assessment tools.

### 2.1 HR-pQCT fracture prediction by correlations in microarchitecture to fracture outcomes

Following the introduction of HR-pQCT in 2005, Boutroy and colleagues showed that HR-pQCT-derived measures (trabecular separation and trabecular bone density) differed between osteopenic women with (n =35) and without a history of fracture (n = 78), even when their aBMD was similar (104). This work was novel, but limited by sample size (104). Still, these findings provided early evidence that HR-pQCT could provide enhanced sensitivity for skeletal fragility than DXA. Boutroy and colleagues proposed that assessments of bone microarchitecture could improve fracture risk prediction, which was confirmed by subsequent studies in cohorts of men and women (26, 105–108). These fracture risk modeling studies largely relied on traditional statistical approaches, including Cox proportional hazards models and logistic regressions to assess the predictive relationships between HR-pQCT parameters and fracture outcomes. Of these studies, Samelson and colleagues presented a comprehensive multi-national study with 7,254 individuals (66% women); this work confirmed that deficits in trabecular and cortical bone density and microarchitecture contribute to fracture risk independently of aBMD and FRAX scores (105). The strongest HR-pQCT predictors of fracture were cortical density, trabecular number, and trabecular thickness at the distal radius (105). At the tibia these same measures, along with cortical area, were identified as the strongest association to fracture. The inclusion of these HR-pQCT measures in fracture risk prediction tools could improve the identification of individuals at risk of experiencing a fracture.

Many HR-pQCT studies have derived significant population-level associations between HR-pQCT measures and fracture outcomes. However, fracture risk prediction tools are intended to provide risk estimates at the individual level, meaning that population-level predictor relationships must be translated into models that can estimate an individual’s fracture risk. This highlights the need for prospective models that can incorporate HR-pQCT parameters into individualized fracture risk assessments. To advance HR-pQCT-based fracture prediction beyond regression models and toward individual fracture assessment, emerging work and novel prediction tools often utilize machine learning (109).

### 2.2 μFRAC: direct assessment of bone in fracture risk prediction

The Microarchitecture Fracture Risk Assessment Calculator (μFRAC) was introduced in 2023, which outputs 5- and 10-year risk of MOF and any osteoporotic fracture (AOF), with AOF defined as any broken bone caused by osteoporosis (99). Inputs include standard HR-pQCT microarchitecture variables of the radius and tibia, vBMD and finite element analysis (FEA) estimations of failure load of the radius and tibia, demographic and anthropometric data (sex, age, height, weight), adulthood fracture history and optionally, DXA-derived aBMD of FN.aBMD (**Table 1**). Standard HR-pQCT parameters of the tibia and radius input into μFRAC include the following: total, cortical, and trabecular BMD; total, cortical, and trabecular cross-sectional areas; derived cortical thickness; trabecular number; and trabecular inhomogeneity. These parameters have previously been described in the literature (100). While the simplified μFRAC″ model requires only age and standard HR-pQCT parameters, the complete μFRAC model integrates additional information like failure load, sex, height, weight, and prior adulthood fracture to improve predictive performance.

The μFRAC tool was created as part of a multi-national collaboration that comprised the data into the Bone Microarchitecture International Consortium (BoMIC, n = 6,802 individuals with mean age of 67.9 ± 8.8, 71% women). To produce fracture risk scores, μFRAC uses a random survival forest algorithm, a supervised machine learning approach that models risk over time (99). Consequently, μFRAC integrates right-censored data to model time-to-fracture outcomes which is in line with traditional statistical tools like Garvan and FRAX. This allows individuals who did not fracture during the study period to remain in the analysis, as they still confer valuable information for distinguishing between individuals who fracture and those who remain fracture-free.

Unlike the established fracture risk assessment tools discussed in Section 1, μFRAC relies on HR-pQCT-derived microarchitecture and vBMD inputs that directly characterize bone (99). As discussed, many existing fracture risk assessment tools rely on CRFs in lieu of direct bone measurements; this reliance can decrease their reliability due to limitations with patient recall, subjective interpretation, and incomplete medical records that may result in a misrepresentation of true fracture risk. Since μFRAC utilizes direct measure of bone architecture and estimated bone strength from FEA, its vulnerability to these issues is decreased. Three variations of μFRAC were developed so that it can be used with or without FEA-based failure load and/or DXA aBMD, meaning that only HR-pQCT parameters and basic CRFs (i.e., age and sex) are required to generate fracture risk scores. Moreover, because μFRAC primarily relies on HR-pQCT-derived microarchitecture and vBMD inputs, it is sensitive to longitudinal changes in bone structure and fracture risk (110). This is in contrast to currently established fracture risk assessment tools that may not be sensitive to adjusted risk when an individual is taking bone-building medication. Tracking changes in risk by µFRAC is useful for monitoring disease progression as well as treatments that affect bone microarchitecture (i.e., pharmacological, diet, exercise, etc.).

Validation of μFRAC has been performed on two independent cohorts to date (111, 112), demonstrating that μFRAC generalizes well to these cohort populations. These validation studies have also highlighted that the fracture discrimination capabilities of μFRAC are strongest for 5-year risk predictions of MOF and AOF compared to 10-year risk predictions. Validation on the Osteoporotic Fractures in Men cohort (MrOS, n =1,586 men, average age 84.4 ± 4.2 years) revealed an AUC of 0.692 (95% CI: 0.686-0.698) for 5-year MOF discrimination, and 0.659 (95% CI: 0.655-0.664) for 5-year AOF discrimination (113). Comparably, in the SUPERB cohort of Swedish women (n = 2,307, average age 77.7 ± 1.6 years), μFRAC produced 5-year MOF scores with an AUC of 0.644 (95% CI: 0.641-0.648) and 5-year AOF scores with an AUC of 0.639 (95% CI: 0.636-0.642) (39). In both external validation studies (MrOS and SUPERB), 5-year MOF and AOF discrimination were similar, although modestly lower than the AUCs observed during initial μFRAC testing with 100-fold cross-validation in the BoMIC cohort (99). On the BoMIC cohort, μFRAC produced 5-year AUCs of 0.714 (95% CI: 0.708-0.719) for MOF and 0.668 (95% CI: 0.664-0.672) for AOF, indicating that model discrimination was broadly comparable across the cohorts for 5-year fracture risk prediction, but slightly reduced outside the development cohort.

A comparison between FRAX with aBMD inputs and μFRAC for 10-year MOF discrimination was also completed in both validation cohorts. In the MrOS cohort, μFRAC had modest yet significant improvements in performance over FRAX with aBMD for 10-year risk of MOF (p<0.05), with μFRAC and FRAX achieving an AUC of 0.687 (95% CI: 0.682-0.692) and 0.613 (95% CI: 0.609-0.617), respectively (113). In the SUPERB validation study, modest yet significant differences in 10-year MOF discrimination were also observed; μFRAC produced an AUC of 0.637 (95% CI: 0.634-0.640) while FRAX with aBMD produced an AUC of 0.628 (95% CI: 0.625-0.632) (39). Ultimately, FRAX and μFRAC performed similarly in the external validation cohorts for 10-year MOF (**Table 3**), even despite differences in their technology and modeling approaches.

Though μFRAC demonstrates modest improvement over FRAX in fracture discrimination, there is significant room for further refinement. Like many fracture risk assessment tools outlined in this review, the discrimination abilities of μFRAC often range between 0.6-0.7, which is considered suboptimal discrimination (39). However, in all instances where μFRAC AUC scores were suboptimal in the SUPERB and MrOS validation studies, μFRAC demonstrated greater risk discrimination compared to reference and FRAX models. Notably, μFRAC showed acceptable-to-moderate discrimination (0.70 or greater) for 5-year risk of MOFs (39), which is likely due to the time frame (4.7 ± 2.4 years) that most of the BoMIC studies followed their participants. Finally, although μFRAC demonstrated statistically significant improvements in fracture discrimination compared to FRAX, the magnitude of these gain were modest (39, 113). Incorporating additional CRFs, such as fall risk, could further refine the μFRAC model and improve fracture discrimination, but this requires further testing.

Several limitations of μFRAC make its clinical practicality challenging. For instance, μFRAC does not produce hip fracture risk scores (114), which will be important to develop in the future given the high clinical burden associated with osteoporotic hip fractures. Another limitation of µFRAC is that it is solely intended for fracture risk assessment, whereas tools such as FRAX can be used as a screening tool. Furthermore, scarcity of HR-pQCT systems makes μFRAC less practical for fracture prediction, with most systems located in research facilities (115). Given the modest benefits in fracture risk prediction, it is currently challenging to justify an increased adoption of HR-pQCT-based approaches; however, it may be well suited for edge cases. For example, when predicted outcomes are borderline using tools like FRAX, μFRAC can provide an additional perspective to support clinical decision-making. As HR-pQCT increases in availability, the comparable performance of μFRAC with other fracture risk tools suggests it has potential as an alternative to DXA-based risk assessment in selected cases.

### 2.3 HR-pQCT and machine learning for hip fracture prediction

Jaiswal and colleagues’ evaluation of machine-learning and traditional statistical models in HR-pQCT-based fracture prediction has some methodological advantages. Using the SUPERB cohort, they developed supervised machine-learning and traditional Cox regression models to evaluate MOF, AOF, and osteoporotic hip fracture prediction (3,028 Swedish women aged 75–80 years, mean age: 77.8 ± 1.6) (114). Their focus on hip fracture risk targets an outcome with substantial declines in quality of life, making the work especially clinically relevant. Moreover, by integrating both traditional statistical and machine-learning techniques with HR-pQCT data, they identify strategies that support the expansion and development of HR-pQCT fracture risk assessment tools (114). Though a formal fracture risk assessment tool is not created in this work, their exploration of HR-pQCT-based fracture prediction is an important basis for further development in the field.

Four supervised machine learning models were trained: neural networks, Extreme Gradient Boosting (XGBoost), ensemble, and random forest. The models used prospective data of individuals who would be monitored for fracture over an 8-year period. For each model, two versions were created. One was a HR-pQCT-input-based machine learning model, and a second reference model used only standard clinical inputs (age, BMI, clinical risk factors, and FN.aBMD). Two additional Cox regression models were created, with one using CRFs and HR-pQCT parameters, and the other relying solely on CRFs (114).

The findings of Jaiswal and colleagues highlight how direct assessments of bone using HR-pQCT parameters alone does not replace the predictive value of CRFs in fracture prediction. Generally, the reference machine learning models using only CRFs performed well compared to the HR-pQCT-based machine learning models. Compared to their HR-pQCT machine learning counterparts, hip fracture discrimination was modestly improved in the reference XGBoost, notably greater in the random forest reference, and the same in the ensemble reference model. The strongest performing model for hip fracture prediction were the neural networks, with both model versions showing acceptable discrimination (AUC = 0.74 for the reference model, and AUC = 0.75 for the HR-pQCT model). Conversely, MOF prediction showed comparable performance across most models, with machine learning HR-pQCT and reference models ranging from AUC values of 0.60 to 0.62. For MOF prediction at the end of follow-up, Cox regression analysis models demonstrated slightly greater discrimination: the full CRF and HR-pQCT Cox regression model, CRF-only model, and 10-year Sweden-specific FRAX with aBMD, had AUC values of 0.66 (95% CI: 0.57-0.74), 0.65 (95% CI: 0.57-0.74), and 0.64 (95% CI: 0.56-0.73), respectively. Notably, Cox regression models outperformed the machine learning models in MOF prediction (114). Overall, this work demonstrates the importance of CRFs in supporting fracture risk prediction beyond just bone.

As this work is exploratory, a formal fracture risk assessment tool is not yet developed. As a result, direct comparisons between existing fracture risk assessment tools like µFRAC and other tools is not possible. To the best of our knowledge, the developed models are not yet externally validated and may have limited generalizability since they were developed and tested in a narrow demographic (women aged 75–80 years).

### 2.4 HR-pQCT and computer vision in fracture prediction

Lu and colleagues used computer vision to define an additional HR-pQCT variable representing textural density directly from scans (116). Textural information was gathered from tibial scans using a 3D local binary pattern method, which is akin to the textural analysis used in TBS from DXA. This textural information was then combined with standard HR-pQCT parameters in a random forest classifier. The predictive performance of the HR-pQCT textural information classifier was compared with other random forest classifiers using different combinations of inputs, all aimed at identifying individuals at heightened fracture risk. The following six random forest classifier variations were created (1): only FN aBMD (2), only CRFs (age, sex, height, weight, BMI, dietary calcium) (3), CRFs and FN aBMD (4), only HR-pQCT image textural information (5), HR-pQCT image texture information and CRFs, and (6) HR-pQCT image texture information with CRFs and aBMD. These models were developed on a relatively small cohort of 345 participants (47.8% women, mean age 76; 97 [28%] with previous fractures). Like Jaiswal and colleagues, this work does not present a formal fracture risk assessment tool but instead broadens the understanding of HR-pQCT and fracture risk assessment beyond Cox regression models (116).

Lu and colleagues work is distinct because it identifies an HR-pQCT textural variable that may have predictive power in fracture risk assessment (116) beyond that of the standard HR-pQCT microarchitectural parameters. In addition, Lu and colleagues’ work found that integrating textural data improved fracture discrimination compared with models including only clinical data and BMD (p < 0.02). Their approach may also capture bone features that CRFs are otherwise used to indirectly represent, indicating that textural information from HR-pQCT may be comprehensive enough to reflect associations of CRFs such as smoking and its impact on bone structure. However, it is unclear whether this information is captured specifically by the textural analysis variable or is already reflected in standard HR-pQCT parameters (116).

The findings of Lu and colleagues indicate that fracture discrimination is driven by their textural HR-pQCT variable. When comparing the random forests model with only (1) FN aBMD and the model (4) with only HR-pQCT textural information, differences in AUC were notable. The AUC for the (1) FN aBMD classifier was 0.66 (95% CI: 0.59-0.73), compared with 0.86 (95% CI: 0.78-0.94) when only (4) HR-pQCT textural information was used for prediction. Overall, the greatest AUC was achieved using the (6) CRFs, FN aBMD, and HR-pQCT textural image data model (AUC: 0.90, 95% CI: 0.83-0.96). When additional CRFs (smoking and physical activity) were integrated into this full fracture assessment model (containing CRFs, FN aBMD, and HR-pQCT textural image data), no statistically significant improvement in AUC was observed. The absence of improved fracture discrimination after adding these CRFs indicates that some relevant information commonly provided by these variables may be captured by HR-pQCT textural data (116).

Although the results of this work are promising, there are some limitations. Lu and colleagues describe how textural information improved fracture discrimination in their logistic regression models more than any given individual HR-pQCT parameter (116). However, this data was not included in the publication, and it is unclear whether the combination of several HR-pQCT parameters can provide the same information conferred by their textural variable. Moreover, the models presented in this work were developed on a small sample size of 345 participants, which increases the risk of overfitting, reduces the stability of performance estimates, and limits the generalizability of the trained classifiers for broader populations. Some of the models presented by Lu and colleagues also require many resources, but their more streamlined versions minimize the need for these resources while still improving fracture prediction (116). Finally, in contrast to the prospective studies by Jaiswal and colleagues (114) and Whittier and colleagues (99), this retrospective work requires independent testing in prospective populations.

## 3 Future directions in fracture risk assessment and current gaps

Approaches for furthering the role of imaging in fracture risk assessment beyond T- and Z-scores include technologies like vertebral fracture assessment (VFA), Radiofrequency Echographic Multi Spectrometry (REMS), opportunistic computed tomography (CT), and HR-pQCT. Extending the role of DXA in quantifying fracture risk is VFA, which the ISCD recommends interpreting using the Genant visual semiquantitative method (117, 118). By combining a qualitative assessment of vertebral morphology and a standardized quantification of height loss, VFA can provide insight into fracture risk independent of aBMD (119). For example, a patient with a normal DXA T-score may be reclassified as having an increased fragility fracture risk if VFA identifies a vertebral fracture (118, 119).

Another technology that can improve understanding of fracture risk is REMS, an ultrasound modality (120) that outputs a Fragility Score (FS) from 0–100 using lumbar and femoral microstructural details (121). A larger FS value is associated with increased bone fragility and fracture risk, with recent work proposing that FS values could be interpreted using intervention thresholds (121). Similar intervention thresholds are used in opportunistic CT-based fracture risk assessment. VirtuOst uses already acquired CT scans that include the hip or spine and FEA to evaluate bone failure loads and fracture risk (122, 123). VirtuOst compares the estimated bone strength against FDA-cleared and statistically derived intervention thresholds, which enables the categorization of individuals at increased fracture risk (123), akin to how BMD-defined osteoporosis identifies individuals at increased fracture risk (124). An emerging advancement in opportunistic CT is the use of deep learning to detect vertebral fractures (125). The integration of radiomics and computer vision has allowed the extraction of features associated with fracture risk that may support fracture risk prediction (126). Relevantly, systematic reviews and meta-analyses have found these models exhibit good discrimination and sensitivity (125, 126), but these technologies are not yet widely available (125). As publications reviewing VFA (118, 119), REMS (127), and opportunistic CT applications (125, 126) for fracture risk prediction are already available elsewhere, this review focuses on HR-pQCT.

Increasingly, HR-pQCT-based fracture risk assessment approaches are being developed that predict fractures using direct quantification of bone (109, 128). Further motivating this HR-pQCT-based risk assessment are the discordances observed between aBMD and HR-pQCT findings. In endocrine conditions like type 2 diabetes mellitus, HR-pQCT can reveal microarchitectural differences even when DXA will not detect differences in aBMD, though the extent of these findings varies across studies (128). This supports the exploration of HR-pQCT-based tools to determine whether they can improve fracture risk prediction. However, these HR-pQCT approaches are encountering many of the challenges faced by established fracture risk assessment tools (e.g., moderate discrimination). Addressing these limitations will be an important step in developing new fracture risk assessment tools and continuing to refine those that are currently available.

Currently, no fracture risk assessment tool supports monitoring treatment-related changes in fracture risk, despite its probable clinical relevance. Fracture risk assessment tools provide static, one-point assessments to stratify individuals into risk categories, yet individuals are treated and monitored longitudinally to reduce fracture risk over time. Tools like FRAX are not intended to assess individuals who have already received bone-building medication (129), or to monitor how fracture risk changes following treatment. Interestingly, HR-pQCT is well established for collecting detailed measurements of bone microarchitectural features (130). Leveraging these high-resolution parameters in fracture prediction may position HR-pQCT-based fracture assessment tools to capture changes in fracture risk following bone-building medication (131). Relevantly, early work demonstrates that μFRAC can track longitudinal changes in fracture risk due to age-associated changes in bone (110), but it remains untested in capturing treatment-related fracture risk changes. Though no fracture risk assessment tool considers treatment-related changes in fracture risk, its inclusion could support clinical workflows to monitor treatment.

Fracture risk assessment tools often prioritize characterizing the contribution of bone to fracture risk. However, for a fragility fracture to occur, deteriorated bone quality coincides with a low trauma event (e.g., a fall from standing height) that causes the fracture (132). Falls account for over 90% of hip fractures (133), and the majority of fragility fractures arise from falls (134). Importantly, fall history is a significant predictor of fracture and demonstrates a dose-dependent relationship: the more frequently an individual falls, the greater their likelihood of experiencing a fragility fracture (135, 136). Notably, a fall within the preceding four months substantially increases fracture risk in older adults; the magnitude of this increased risk is comparable to that of a recent fracture in predicting future fracture (137). Despite existing literature confirming that falls predict fractures independently of bone, tools like FRAX, CAROC, and μFRAC do not incorporate fall history. QFracture and Garvan incorporate history of falls, but only Garvan includes a dose-based response to fall risk (the number of falls in the previous 12 months). No HR-pQCT-based fracture assessment model discussed in this review integrates fall history.

In addition to fall history, other risk factors for falls are well established and include balance and strength impairment, visual impairment, orthostatic hypotension, polypharmacy and cognitive impairment (138). Specific functional muscle measures may also be important (139), such as hand grip strength (HGS), one-leg standing time (140), the timed-up-and-go (TUG) test and the Berg Balance Scale (141). The motivation for using muscle function as a falls risk factor is that sarcopenia increases fall risk, and tracking muscle may help capture this added risk. Existing work has also examined how functional muscle measures contribute to fracture risk independently of BMD and CRFs, with conflicting results (142).

Among the functional muscle measures discussed above, HGS may be of particular interest because it can be easily measured in the clinical setting and is a measurement that is captured in many large HR-pQCT cohorts (105). The role HGS plays in fall risk is complex (143). For instance, the ability to grip a support and regain an upright position to prevent a fall partly relies on HGS. However, whether HGS is independently associated with fracture risk remains contested. Some studies describe a relationship between HGS and fracture risk (142, 143), whereas others have found no statistically significant association between HGS and observed fracture risk (144). Muscle metrics using DXA, such as muscle cross-sectional area and lean body mass (145), have been included as possible fall risk factors to identify individuals at risk of experiencing fragility fracture; however their role in fracture prediction remains unclear (146). Fall-Risk Inducing Drugs (FRIDs) and polypharmacy have also been shown to increase the risk of fragility fractures (147). While current fracture risk assessment tools account for medications, they assess medications that influence bone strength like glucocorticoids, rather than medications that increase the risk of fracture through a greater likelihood of falling, such as benzodiazepines. A model that combines HR-pQCT-based characterization of bone with fall risk factors warrants consideration. Existing prospective datasets like BoMIC (105) and MrOS (112) are opportunities to develop this type of model, as they contain HR-pQCT outcomes, fall frequency in the previous 12 months, and other risk factors for falls, including functional muscle measures.

The inclusion of fall risk factors in fracture risk assessment tools will require that the resulting fracture risk can be interpreted in the clinical setting. Identifying how fall risk factors and bone independently contribute to overall fracture risk is necessary for targeted intervention. If bone is the primary driver of an individual’s fracture risk, this should be clearly identifiable to ensure patients receive appropriate treatments like bone-building medications. Similarly, identifying the individual’s risk factors that contribute to falls risk will also support targeted interventions. There is robust evidence to suggest that strength and balance training, as well as multifactorial interventions (i.e., interventions that simultaneously target multiple risk factors), are effective in preventing falls in targeted populations (148, 149).

Several challenges in fracture risk assessment tool development are unique to HR-pQCT. For example, if lean mass from a standard DXA scan is shown to be an important factor in fracture prediction, HR-pQCT-based prediction tools may be limited because HR-pQCT does not capture this measure. Acquiring and quantifying muscle measures is challenging at peripheral scanning sites, as primarily tendinous tissue is found in the standardized HR-pQCT ultra-distal measurement regions. Additionally, scanner availability remains a challenge for any HR-pQCT-based tool. Even for HR-pQCT-based approaches that demonstrate comparable or superior performance in fracture risk discrimination (99, 114, 116), their broader implementation depends on scanner access and clinical familiarity. DXA, however, remains widely used in part because it is already integrated within existing fracture risk assessment tools and clinical workflows. Therefore, the potential value of HR-pQCT-based tools will necessarily be considered alongside practical barriers to adoption, including cost, availability, and integration with existing clinical workflows. A key question that remains unanswered is whether improved fracture risk stratification warrants the economic investment required for wider HR-pQCT clinical use. Given the high cost of osteoporosis and fragility fractures, improved identification of individuals at risk of fracturing, followed by targeted intervention to prevent fractures, may have economic benefits; however, a formal analysis is required, and continual improvement of HR-pQCT fracture risk models is needed.

## 4 Conclusion

Established and emerging fracture risk assessment tools incorporate different modeling approaches, assumptions, and data to stratify fracture risk, creating advantages and challenges for each tool. The performance of these tools often borders clinical utility, with the majority demonstrating moderate discrimination (AUC values below 0.80) (**Tables 2** and **3**). Despite this, many fracture risk assessment tools still demonstrate a superior ability in identifying individuals at risk of osteoporotic fracture compared to clinical standards of using reference cohort-based assessments. Common fracture risk assessment tools like Garvan and FRAX demonstrate improved risk prediction using DXA-derived aBMD and CRFs, compared to risk stratification with DXA and reference data alone. Interestingly, emerging approaches in HR-pQCT-based fracture risk assessment demonstrate fracture discrimination that is consistently as good as, and at times better than, that of these established tools. Although emerging tools require further development and are clinically limited by HR-pQCT availability, their fracture discrimination is promising and warrants further research. Future work should also consider exploring how fracture risk assessment tools reclassify individuals across risk strata (40), and how the inclusion of factors such as fall risk, which independently contribute to fracture risk, may improve the performance of established and emerging tools. Finally, future research would also likely benefit from large-scale collaborations, like BoMIC, to support tool development and comparisons between fracture risk assessment tools.
